# Impact of the COVID-19 Pandemic on the Management of *Staphylococcus aureus* Bloodstream Infections in a Tertiary Care Hospital

**DOI:** 10.3390/pathogens12040611

**Published:** 2023-04-18

**Authors:** Christian W. Böing, Neele J. Froböse, Frieder Schaumburg, Stefanie Kampmeier

**Affiliations:** 1 Institute of Hygiene, University Hospital Münster, Robert-Koch-Straße 41, 48149 Münster, Germany; christian.boeing@ukmuenster.de; 2 Institute of Medical Microbiology, Domagkstraße 10, 48149 Münster, Germany; neelejudith.froboese@ukmuenster.de (N.J.F.); frieder.schaumburg@ukmuenster.de (F.S.)

**Keywords:** *Staphylococcus aureus*, bacteremia, COVID-19, antimicrobial stewardship

## Abstract

*Staphylococcus aureus* bacteremia (SAB) is associated with a high mortality rate. The clinical outcome of SAB patients highly depends on early diagnosis, adequate antibiotic therapy and source control. In the context of the COVID-19 pandemic, the health care system faced additional organizational challenges and the question arose whether structured screening and triaging for COVID-19 and shifting resources influence the management of SAB. Patients (*n* = 115) with SAB were enrolled in a retrospective comparative study with historical controls (March 2019–February 2021). The quality of SAB therapy was assessed with a point score, which included correct choice of antibiotic, adequate dosage of antibiotic, sufficient duration of therapy, early start of therapy after receipt of findings, focus search and taking control blood cultures 3–4 days after starting adequate antibiotic therapy. The quality of treatment before and after the onset of the COVID-19 pandemic were compared. No significant differences in the total score points were found between the pre-COVID-19 and COVID-19 cohort. All quality indicators, except the correct duration of antibiotic therapy, showed no significant differences in both cohorts. Furthermore, there were no significant differences in the outcome between both cohorts. The treatment quality of SAB therapy was comparable before and during the COVID-19 pandemic.

## 1. Introduction

*Staphylococcus aureus* bacteremia (SAB) is a severe invasive infection which is still associated with a high mortality of up to 27% in the first three month after diagnosis [1]. The prevalence of SAB in Europe is up to 19.5% among nosocomial acquired bacteremia [2]. The ratio between nosocomial and community-acquired SAB is slightly in favor of nosocomial cases at 51% [3]. Intravascular catheters, skin and soft tissue infections, endocarditis, osteomyelitis, post-operative wound infections or respiratory infections are common foci of SAB [4,5,6].

The outcome of SAB is affected by many factors: mortality depends strongly on the age of the patients, as the mortality rate seems to increase by about 1.3 times every 10 years. In addition, the presence of shock symptomatology at the time of diagnosis and the focus of SAB appear to play a role in the outcome of SAB [7].

Persistence of *S. aureus* in the blood and resulting secondary metastatic infections can be the consequence of inadequate therapy [8,9,10]. The treatment of SAB requires compliance with certain treatment standards that can significantly reduce mortality and improve the outcome of the patient [8,11,12,13,14]. Quality features of SAB therapy include the choice of an adequate antibiotic and an adequate dosage and duration of administration, as well as the earliest possible start of antibiotic therapy [1,11,15], a focus search and, at best, its remediation [13].

The implementation of antimicrobial stewardship (AMS) programs could significantly improve the treatment and outcome of SAB. Classical aspects of an AMS intervention include monitoring microbiological diagnostics or diagnostic findings, selection of adequate antibiotic therapy, duration of therapy and focus search [16].

With the start of the COVID-19 pandemic, the health system was confronted with new challenges, especially organizational ones, which made it more difficult to comply with and implement existing treatment standards. In patients presenting to hospitals with fever, COVID-19 is considered an additional differential diagnosis since then; in the case of underlying SAB, the diagnostic focus may initially be shifted and organizational measures regarding COVID-19 may be taken until COVID-19 is ruled out. The objective of this study was to test if the quality of SAB management deteriorated during the COVID-19 pandemic compared to a pre-COVID-19 control group.

## 2. Materials and Methods

### 2.1. Clinical Setting and Study Design

The study was conducted at the University Hospital Münster (UHM), a 1500-bed tertiary care hospital admitting approximately 55000 patients per year. During a 2-year time-period (March 2019–February 2021), all patients with SAB were included in our study. Exclusion criteria were: (i) polymicrobial bacteremia, (ii) death or discharge within 5 days after initial positive blood culture, (iii) admission from another hospital with already diagnosed SAB. As this study investigates the impact of routine AMS visits on quality of care and (iv) patients who had an infectious disease consultation in response to SAB detection were excluded (Supplementary Figure S1). Basic patient characteristics including demographics, underlying comorbidity, primary focus of infection, AMS consultation and antibiotic resistance were documented. Nosocomial SAB was defined as onset of symptoms > 48 h after admission of the patient. Cases between March 2019–February 2020 were assigned to the pre-COVID-19 cohort; cases during March 2020–February 2021 were included in the COVID-19 cohort. Quality of treatment was assessed by six quality indicators [17] which comprised the (i) correct choice of antibiotic (the narrow ß-lactams flucloxacillin or cefazolin in cases of methicillin sensitive *S. aureus* (MSSA) or vancomycin in cases of methicillin resistant *S. aureus* (MRSA), both in the absence of allergies to the corresponding substances), (ii) adequate dosage of antibiotic (flucloxacillin 12 g/d divided into 3 to 6 single doses, cefazolin 3 × 2 g/d, adapted to renal function; vancomycin serum trough level determined by therapeutic drug monitoring [target: 10–20 mg/L]), (iii) sufficient duration of therapy (at least 14 days for uncomplicated SAB and 28 days for complicated SAB), (iv) early start of therapy after receipt of results via phone call by a physician of the Medical Microbiology department (<24 h), (v) adequate focus search (e.g., transthoracic/transesophageal echocardiography, abdominal sonography, other imaging and microbiological examination of devices) and (vi) taking control blood cultures 3–4 days after start of adequate antibiotic therapy to check for persistent bacteremia. The total quality of care for every patient was assessed by a score system: the presence of each quality indicator was included with one score point into the score system; hence, the minimum total score was 0 and the maximum total score was 6 points.

Diagnostic consultation of the treating department took place on the one hand through consultation by the physicians of the Medical Microbiology department at the time of the initial findings by telephone call, and on the other hand in certain departments additionally through an AMS team within the framework of a weekly treatment visit to the ward. The prognostic outcome of the SAB treatment was assessed by persistent bacteremia of ≥3 days.

### 2.2. Blood Culture Sampling

Aerobic/anaerobic blood cultures (Bactec, BD, Heidelberg, Germany) that were flagged positive within the described investigation time period and showed growth with *S. aureus* were included. Included blood cultures were microscopically examined after Gram staining. Two drops of blood culture broth were spread on Columbia blood agar (BD) and incubated at 5% CO_2_ and 36 ± 1 °C. Species identification was performed with matrix-assisted laser desorption/ionization time of flight mass spectrometry (MALDI-TOF MS) measurement using the Microflex instrument (Bruker, Bremen, Germany) after short incubation period of three hours [18]. Phenotypic resistance testing was performed using Vitek 2 automated system (bioMérieux, Marcy l’Étoile, France) and interpreted according to the EUCAST clinical breakpoints of the respective years.

### 2.3. Statistical Analysis

Descriptive statistics were expressed by total numbers and percentages for categorical variables. Univariable analysis was performed using the Chi-squared test for categorical variables. Odds ratios (OR) and 95% confidence intervals were calculated for each categorical variable. Mann–Whitney U test was perform to compare non-parametric data. A *p*-value < 0.05 was considered statistically significant. Statistical analysis was performed using R Studio version 1.3.1056 (R version 3.6.3) (The R Foundation, Vienna, Austria).

### 2.4. Ethics Approval

The study was approved by the institutional review board (IRB, Ethikkommission der Westfälischen Wilhelms-Universität Münster, file number 2021-538-f-S, 3 September 2021) and was carried out in accordance with relevant guidelines and regulations.

## 3. Results

### 3.1. Patient Characteristics and Outcome Parameters

In total, 156 patients were recruited for this study. After application of our exclusion criteria, 115 cases of SAB were eligible for analysis. Forty-one patients were excluded because nine patients received extensive consultation by an infectious disease specialist, seventeen died or were discharged within five days, five patients were admitted from another hospital with SAB and ten patients were excluded because of missing data concerning antibiotic therapy. Of the included cases, 48 belonged to the pre-COVID-19 cohort and 67 to the COVID-19 cohort. The basic patient characteristics of both cohorts, including demographic data, aetiology of SAB, severity of existing underlying disease (assessed by the Charlson comorbidity index and Pitt bacteremia score) and MRSA prevalence, were not significantly different (Table 1). Two patients in the COVID-19 cohort were infected with SARS-CoV-2 while suffering from SAB. Patients in the pre-COVID-19 cohort had a higher number of unknown foci compared to the COVID-19 cohort (31% vs. 16%). The most dominant focus in both cohorts was intravascular devices (pre-COVID-19: 19% vs. COVID-19: 39%). The COVID-19 cohort had a higher prevalence of native valve endocarditis, intravascular device infection, skin and soft tissue infection and surgical site infection (Table 1). AMS consultations were slightly and not significantly more prevalent in the pre-COVID-19 than in the COVID-19 cohort (46% vs. 36%, *p* = 0.280). The rate of persistent bacteremia was not significantly different in the two cohorts (13% vs. 13%, *p* = 0.958). The time interval between the first onset of symptoms and the initial collection of blood cultures was not significantly different between the two cohorts (median = 0 days vs. 0 days, *p* = 0.606).

### 3.2. Quality of Care Score

**Figure 1:**
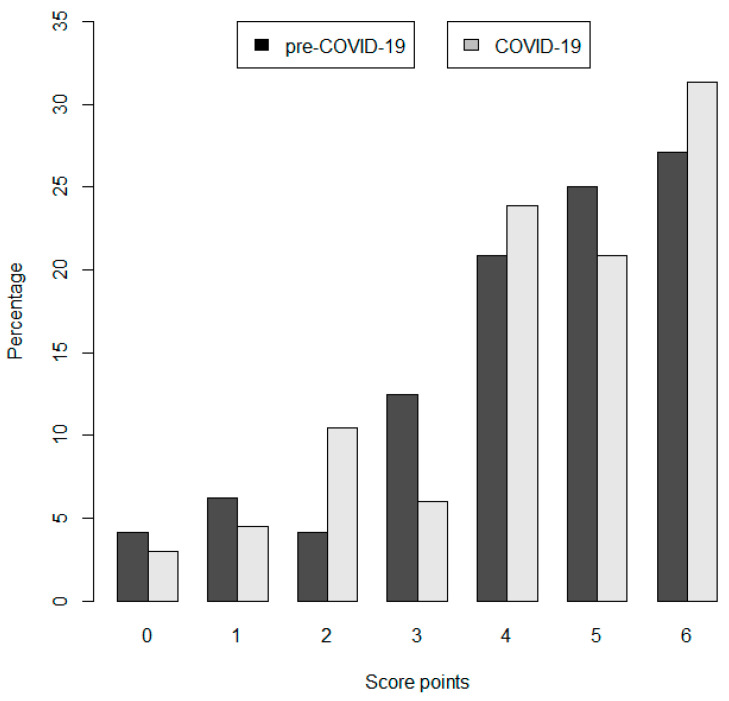
Relative proportion of maximum total score points in the pre-COVID-19 and COVID-19 cohort.

No significant differences in the quality indicators were found in both cohorts except the correct duration of antibiotic treatment, which was more often implemented in the COVID-19 cohort (66% vs. 86%, *p* = 0.019). Table 2 displays the results of the fulfilled quality indicators. The comparison of the total score points of both cohorts showed also no significant difference, *p* = 0.76 (Figure 1). Patients who were visited by the AMS team (*n* = 46) had significantly higher score points than patients who were not visited by the AMS team (*n* = 69) (median = 4 vs. 5; *p* = 0.002).

## 4. Discussion

In this study, we investigated the impact of the COVID-19 pandemic on the management of SAB treatment, a severe invasive infection with high morbidity and mortality. Our focus was to elucidate whether the COVID-19 pandemic had an impact on the quality of care of SAB regarding the immediate diagnostic and therapeutic management in a maximum care hospital.

Collateral damage of the COVID-19 pandemic with a deterioration in treatment quality and prognosis have been documented for several diseases such as strokes and myocardial infarction [19,20]. So far, the impact of COVID-19 on infectious diseases has been scarcely investigated. There is evidence that the COVID-19 pandemic had a negative impact on hygiene management with the consequence of multi-drug resistant organism (MDRO) outbreaks and spread of MDROs [21]. Reduced adherence to hygienic measures, especially hand hygiene, could also explain the increased rate of catheter-associated infections in the COVID-19 cohort in our study. However, our results do not suggest a deterioration in the overall quality of care of patients with an SAB during the COVID-19 pandemic. We found neither a significant difference between the total score points of both cohorts nor a worsening of prognostic outcome and therapy failure in the COVID-19 cohort. This is in particular remarkable as other clinical disciplines experienced significant impairments in the diagnosis and treatment of diseases during the first waves of the COVID-19 pandemic [22,23,24]. On the one hand, these different observations might be due to biased awareness of healthcare professionals in favor of infectious diseases. On the other hand, restrictions on elective interventions and treatments as a result of intensified hygiene measures during the pandemic can be responsible for these findings.

This consistency in the quality of treatment can be explained by our diagnostic and therapeutic approach in case of SAB. As part of our routine procedure, the diagnosis of SAB is always immediately communicated by the physicians of the Medical Microbiology department to the treating colleagues. In addition, both therapeutical and further diagnostic advice, such as infectious source control and taking further blood cultures, was given in the laboratory report. A large proportion of SAB patients were also visited by the AMS team. Hence, adherence to treatment standards was routinely ensured through consultation with an interdisciplinary team of experts. We found that patients that were visited by the AMS team had significantly higher score points than patients without AMS consultation. This underlines the importance and relevance of infectious disease counselling by appropriate experts in the field of medical microbiology or AMS during the COVID-19 pandemic [25].

Besides the consistent treatment quality, no impairment in prognostic outcome was found. The outcome of most acute severe disease that showed an impaired prognosis during the COVID-19 period, i.e., strokes and myocardial infarction, depended on pre-hospital factors, intrahospital diagnosis and treatment quality. In more than two-thirds of the patients in our study, SAB (pre-COVID-19: 70%, COVID-19: 70%) was acquired nosocomially. The pre-hospital and patient related prognostic factors, especially higher threshold seeking health care and delayed admission to hospital because of fear of SARS-CoV2 infection that were associated with impaired prognosis, may be less important in SAB patients [26].

This study has limitations: (i) the local area of Münster had relatively low numbers of COVID-19 cases in the first and second waves of the pandemic compared to other European countries that coincided with the survey period of the COVID-19 cohort [27]. Our findings can therefore not be transferred to high incidence areas of COVID-19 with a massive burden of disease for the health care facilities; (ii) low case numbers, especially in the pre-COVID-19 cohort, may influence the results due to lower test power; and (iii) our analysis refers exclusively to the SAB case numbers and therapy in a maximum-care hospital and is therefore a single-center study. Additional multi-center studies in acute care hospitals or data from areas with higher incidence of COVID-19 cases are necessary to prove the hypothesis of whether there has been a deterioration in the diagnostic and treatment management of SAB during the pandemic.

## 5. Conclusions

Although the COVID-19 pandemic poses major challenges to hospitals in the diagnosis and care of other infectious diseases, this study did not demonstrate any impairment in treatment quality in patients with SAB.

## Figures and Tables

**Table 1:** Characteristics of patients with *S. aureus* bacteremia in the pre-COVID-19 and COVID-19 cohort.

Characteristics	Pre-COVID-19 Cohort (*n* = 48)	COVID-19 Cohort (*n* = 67)	*p*-Value
Demographics			
Male sex [*n* (%)]	34 (71)	43 (64)	0.454
Median age (IQR *)	67.5 (28)	61 (29)	0.180
Acquisition			
Nosocomial [*n* (%)]	33 (70)	47 (70)	0.994
Underlying disease severity			
Charlson comorbidity index [median (IQR *)]	2 (2)	2 (3)	0.257
Median Pitt bacteremia score [median (IQR *)]	1 (2)	1 (1.5)	0.662
Focus			
Bone and joint infection [*n* (%)]	2 (4)	1 (1)	-
Deep tissue infection [*n* (%)]	4 (8)	0 (0)	-
Foreign body [*n* (%)]	1 (2)	0 (0)	-
Intravascular device [*n* (%)]	9 (19)	26 (39)	-
Native valve endocarditis [*n* (%)]	1 (2)	6 (9)	-
Other focus [*n* (%)]	2 (4)	2 (3)	-
Pneumonia [*n* (%)]	4 (8)	6 (9)	-
Prostethic valve endocarditis [*n* (%)]	1 (2)	2 (3)	-
Prosthetic joint [*n* (%)]	2 (4)	0 (0)	-
Skin and soft tissue infection [*n* (%)]	2 (4)	7 (10)	-
Surgical site infection [*n* (%)]	3 (6)	6 (9)	-
Urogential [*n* (%)]	2 (4)	0 (0)	-
Unknown [*n* (%)]	15 (31)	11 (16)	-
AMS consultation [*n* (%)]	22 (46)	24 (36)	0.280
MRSA [*n* (%)]	3 (6)	5 (7)	1

* IQR: inter-quartile range.

**Table 2:** Quality indicators of *S. aureus* bacteremia treatment fulfilled in the pre-COVID-19 and COVID-19 cohort.

Quality Indicator	Pre-COVID-19 Cohort (*n* = 48)	COVID-19 Cohort (*n* = 67)	OR (95%-CI)	*p*-Value
Correct antibiotic agent [*n* (%)]	41 (85)	57 (85)	0.97 (034–2.77)	0.959
Early therapy (<24 h) [*n* (%)] *	30 (75) **	42 (74)	0.93 (0.37–2.36)	0.884
Dosing [*n* (%)] *	33 (80)	43 (75)	0.74 (0.28–1.98)	0.555
Therapy duration [*n* (%)] *	27 (66)	49 (86)	3.18 (1.18–8.53)	0.019
Focus search [*n* (%)]	38 (79)	46 (69)	0.58 (0.24–1.37)	0.210
Follow-up blood cultures day 3/4 [*n* (%)]	34 (71)	52 (78)	1.43 (0.61–3.33)	0.409

* values refer exclusively to cases that received a correct antibiotic. ** start of antibiotic therapy was not documented in one case.

## Data Availability

All relevant data analyzed during this study are included in this article.

## Supplementary Materials

The following supporting information can be downloaded at: https://www.mdpi.com/article/10.3390/pathogens12040611/s1, Figure S1: SAB patients meeting the inclusion criteria.
