# Supplementary figures and images for: Impact of the COVID-19 Pandemic on the Management of Staphylococcus aureus Bloodstream Infections in a Tertiary Care Hospital
Source: Pathogens. 2023 Apr 18;12(4):611. doi: 10.3390/pathogens12040611 (PMC10143185; doi:10.3390/pathogens12040611)

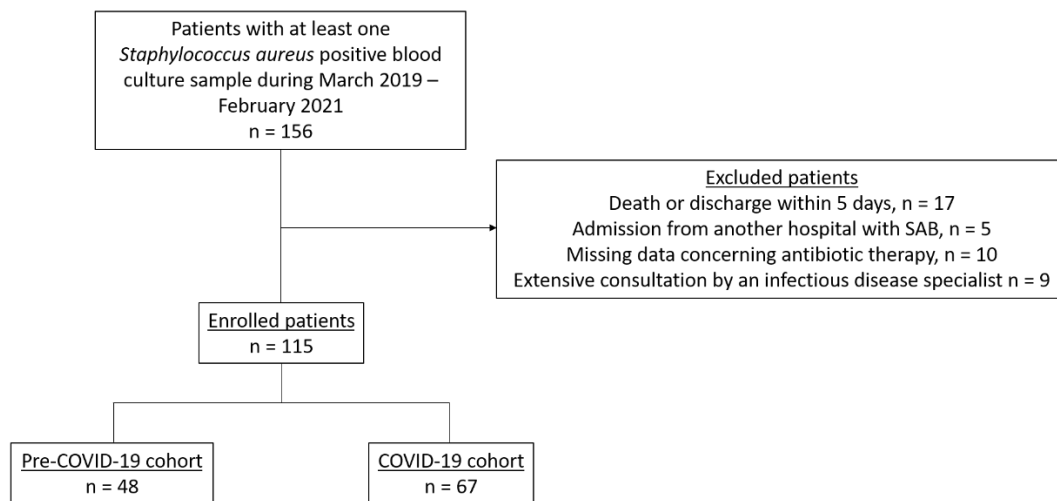

**Supplementary Figure S1.** SAB patients meeting the inclusion criteria

Supplement: Supplementary file 1 [file pathogens-12-00611-s001.zip › pathogens-2333766-supplementary.pdf]
